# Sexual and Reproductive Health Knowledge, Contraception Uptake, and Factors Associated with Unmet Need for Modern Contraception among Adolescent Female Sex Workers in China

**DOI:** 10.1371/journal.pone.0115435

**Published:** 2015-01-27

**Authors:** Megan S. C. Lim, Xu-Dong Zhang, Elissa Kennedy, Yan Li, Yin Yang, Lin Li, Yun-Xia Li, Marleen Temmerman, Stanley Luchters

**Affiliations:** 1 Centre for Population Health, Burnet Institute, Melbourne, Victoria, Australia; 2 International Centre for Reproductive Health, Department of Obstetrics and Gynaecology, Ghent University, Ghent, Belgium; 3 Centre for International Health, Burnet Institute, Melbourne, Victoria, Australia; 4 Department of Epidemiology and Preventive Medicine, School of Public Health and Preventive Medicine, Monash University, Melbourne, Victoria, Australia; 5 School of Public Health, Kunming Medical University, Kunming, China; 6 Department of AIDS Management, Kunming the Third People’s Hospital, Kunming, China; 7 Department of Primary Health Care, Kunming Maternal and Child Health Care Centre, Kunming, China; 8 Department of AIDS Prevention, Kunming Maternal and Child Health Care Centre, Kunming, China; University of Aberdeen, UNITED KINGDOM

## Abstract

**Objective:**

In China, policy and social taboo prevent unmarried adolescents from accessing sexual and reproductive health (SRH) services. Research is needed to determine the SRH needs of highly disadvantaged groups, such as adolescent female sex workers (FSWs). This study describes SRH knowledge, contraception use, pregnancy, and factors associated with unmet need for modern contraception among adolescent FSWs in Kunming, China.

**Methods:**

A cross-sectional study using a one-stage cluster sampling method was employed to recruit adolescents aged 15 to 20 years, and who self-reported having received money or gifts in exchange for sex in the past 6 months. A semi-structured questionnaire was administered by trained peer educators or health workers. Multivariable logistic regression was conducted to determine correlates of low knowledge and unmet need for modern contraception.

**Results:**

SRH knowledge was poor among the 310 adolescents surveyed; only 39% had heard of any long-acting reversible contraception (implant, injection or IUD). Despite 98% reporting not wanting to get pregnant, just 43% reported consistent condom use and 28% currently used another form of modern contraception. Unmet need for modern contraception was found in 35% of adolescents, and was associated with having a current non-paying partner, regular alcohol use, and having poorer SRH knowledge. Past abortion was common (136, 44%). In the past year, 76% had reported a contraception consultation but only 27% reported ever receiving SRH information from a health service.

**Conclusions:**

This study demonstrated a low level of SRH knowledge, a high unmet need for modern contraception and a high prevalence of unintended pregnancy among adolescent FSWs in Kunming. Most girls relied on condoms, emergency contraception, or traditional methods, putting them at risk of unwanted pregnancy. This study identifies an urgent need for Chinese adolescent FSWs to be able to access quality SRH information and effective modern contraception.

## Introduction

Adolescents constitute a large and important target population for sexual and reproductive health (SRH) interventions. Onset of sexual activity during adolescence is common, however, young people experience significant barriers that limit their access to essential SRH information and services[1]. Consequently, adolescents suffer a disproportionate burden of poor SRH outcomes, including early and unintended pregnancy. Globally, adolescent girls aged 15–19 years have among the lowest knowledge and use of contraception and the highest unmet need of any age group[2,3]. An estimated 16 million adolescent girls give birth each year, which contributes to morbidity and mortality, low educational attainment and socio-economic disadvantage of girls and their families[3,4,5,6]. Preventing adolescent pregnancy and increasing use of modern contraception is therefore an important public health strategy [6].

Adolescents represented almost 14% of the population in China in 2009[7]. China’s young generation is growing up in a rapidly changing society and in the past two decades increasingly engaging in premarital sex[8–11]. However, complex challenges exist in adequately and effectively meeting the SRH information and service needs of unmarried young people[12,13]. Government subsidised family planning (FP) services are generally only accessible to married couples[9,13,14]. Moreover, traditional socio-cultural taboos regarding premarital sex and pregnancy impede access to SRH services and limits the provision of comprehensive SRH education through schools and other sources[12]. Each year, an estimated ten million induced abortions occur in registered health facilities, excluding self-induced abortion by oral mifepristone and/or misoprostol, with approximately 25% among unmarried women younger than 18 years[13,15]. A 2010 nationally representative survey of nearly 11,000 unmarried women aged 15–24 years[11] and a 2009 study of over 5,000 unmarried women with a mean age of 20 years (SD = 2.2)[16] both showed very low levels of comprehensive SRH knowledge despite 19% and 17% of participants reporting to be sexually-active, respectively. Of those who were sexually-active, 21% and 26% reported premarital pregnancy of which the majority (91%) ended in induced abortion. Young women aged 15–19 years who are out-of school had the lowest level of SRH knowledge. Among sexually-active 15–19 year olds, the prevalence of unprotected sex, multiple abortions, and unsafe abortions exceeded that of women aged 20–24 years[11].

Adolescents are a diverse group whose capacities and needs differ by developmental stage, schooling, social capital and legislative status[17,18]. Despite growing efforts to improve the SRH of unmarried young people in China[8–10,12,16,19], certain disadvantaged groups of adolescents with high need have been neglected, including those engaged in sex work. In China, sex work is illegal and highly stigmatised. The sex industry has been driven by socio-economic pressure for girls who drop out of school, large income disparities among rural and urban areas, limited employment opportunities, sex ratio imbalance (sex ratio at birth was 117.8 boys to every 100 girls in China in 2011) and relaxation of attitudes about sexuality[20–22]. The estimated number of female sex workers (FSW) was 25,000 in 1985, and an estimated 2.8–4.5 million in 2005[23]. In recent years, the number of young FSWs in their teens or early 20s has also risen in large cities and tourist areas of China. Reliable data are difficult to obtain, but experts estimate that between 15 to 40 per cent of the FSWs population is aged under 20 years, similar to other Asian countries[24,25]. There is limited understanding of the unique SRH challenges that young FSWs face in China. These disparities must be addressed when attempting to improve and identify the programmatic implications of SRH among this population.

The aims of this study were to assess sexual and reproductive health knowledge and determine factors associated with low knowledge; to describe use of contraception and condoms; to determine factors associated with unmet need for contraception; to describe pregnancies and their outcomes, and the use of and need for SRH services; and to determine factors associated with experience of violence among adolescent female sex workers in Kunming, China.

## Methods

### Study setting

Yunnan Province, in the southwest of China, is a multi-ethnic area and the largest economic centre and capital of Yunnan province with an estimated population of 6.4 million. Despite sex work being criminalized in China, it is estimated over 10,000 FSWs are active in Kunming, excluding street-based and freelance FSWs [26]. Within all four urban districts of Kunming, most entertainment establishments (e.g., karaoke, night club, dancing hall, disco, bar) or personal service sectors (e.g., hair washing rooms, hair salons, massage parlour, sauna, restaurant, hotel) also provide sex services[27]. Recently, HIV/STIs intervention programs and local FSW community-based organizations have reported an increase in the number of teenage women engaged in sex work, the majority being rural-to-urban migrants[27].

### Study design, participants and sampling procedures

This cross-sectional study was conducted between July and September 2012 in all four urban areas of Kunming. The study population were eligible to participate in the study if they were women aged 15 to 20 years old, currently living in one of the four urban study sites, and who self-reported having received money or gifts in exchange for sex from a paying partner in the past 6 months. No additional exclusion criteria were used.

In collaboration with local Kunming FSW support organizations and the district level Centres of Disease Control (CDC), 101 locations were identified and mapped where young FSWs sell sex in the four urban districts of Kunming. In addition, the peak working hours for these locations were recorded. A one-stage cluster sampling method was employed to recruit study participants. The required sample size of 316 was determined by using formula: n = (1.96)^2 (0.25)(1–0.25)/(0.05)^2 ×110% (assumed adolescent FSWs prevalence rate was set as 0.25%, α = 0.05, power = 0.95, non-response rate as 10%). The initial stage of sampling involved 27 clusters(locations) which were randomly selected from the 101 identified locations, proportionate to the total number of locations in each district. Potentially eligible women were recruited from these 27 clusters proportional to the total number of FSW in each district until the required sample size was recruited from each district.

A semi-structured questionnaire in Chinese was adapted from a WHO survey questionnaire for young people, the Demographic and Health Survey (DHS) youth questionnaire, and a previously used cohort study among sex workers[28,29]. Key informants including leaders of FSWs’ support organizations, senior peer educators and health workers were invited to review each revision of the questionnaire. The questionnaire was pre-tested among 14 young FSWs to ensure the content and language was appropriate for the study population. The questionnaire covered eight domains: socio-demographic information; knowledge and sources of SRH information; ever and current contraceptive use; experience of intimate partner violence (paying and non-paying partners); alcohol and other drug use; self-reported history of sexually transmitted infections (STI) and symptoms; previous pregnancy, pregnancy intentions and outcomes; and health seeking behaviour.

A total of six peer educators (former FSWs from local FSW organizations) and six health workers (doctors/nurses selected from district level CDCs and hospitals) were trained as interviewers on the study procedures and questionnaire administration in a two-day workshop in Kunming. The training focused on i) understanding the background and objectives of the study; ii) sampling strategy and recruitment process; iii) field monitoring process; iv) interviewing skills with sensitive issues including ethical aspects and confidentiality; v) data collection and management. Four teams of three interviewers were established and allocated to each of the four districts.

Interviews were administrated face-to-face at different entertainment venues including at karaoke clubs, nightclubs, dancing halls, discos, bars or personal service sectors (e.g., hair washing room, hair salon, massage parlour, sauna, restaurant, hotel), where young FSWs were working. Where possible, initial permission was sought from managers or owners of entertainment establishments, and an appointment arranged for a visiting time for the purposes of recruitment, while these gatekeepers also introduced the team to potentially eligible women. When requested by participants, some interviews were performed in drop-in centres within FSW support organizations. Interviews took between 40 and 50 minutes to complete.

The paper-based data were double entered using EpiData (version 3.1) by separate trained staff. Following data checking and cleaning, the final dataset was available for analysis.

### Ethical consideration

Ethics approval and permission to the study protocol, informed consent forms and procedures, information sheet and other requested documents, or any subsequent modifications—were obtained from the ethics committee of Kunming Public Health Bureau (study No. KM-FSW-12–01). All interviewers received training in research ethics, including non-judgemental interview skills and confidentiality.

All participants were clearly informed about the study objectives, the confidential nature of information collected, their rights of voluntary involvement, refusing to answer question and withdrawing; and all participants provided their written informed consent before the interview. For those who were under the age of 18 and were living apart from their parents and self-supporting, staff asked participants for the best way to get a written informed consent; and based on the WHO’ suggestion on SRH research among young adolescents in developing countries [30], a formal written informed consent was obtained from an adult peer if indicated by the participant younger than 18 years of age.

Participants were compensated 50RMB (approximately 6EUR) for their time and return transportation. Following the interview, free male condoms, pamphlets about SRH, and information sheets providing details about FSWs support groups, STI clinics and antenatal care clinics were offered to the respondents. In addition, a counselling session on SRH and contraception was provided to participants.

### Measures

Modern contraception was defined as sterilisation, oral contraceptive pill, intra-uterine device (IUD), diaphragm, injection, emergency contraception, or implant. Because almost all adolescents reported using condoms, these were excluded from the definition of modern contraception. This allowed for differentiation between those with higher and lower levels of knowledge. Long-acting reversible contraceptive methods (LARCs) were defined as intra-uterine device (IUD), hormonal injections, or implants. Knowledge of SRH was derived from a set of seven true/false questions (Table 1) regarding methods for preventing pregnancy (one point for each correct answer) plus one point for having heard of each of eight methods of contraception (seven modern methods plus condom) when prompted with a list of methods. All contraception methods were verbalized, and interviewers described the method in detail. The maximum score was15. Scores were split at the median (seven or less vs eight or more) to create a dichotomous low/high knowledge score.

**Table 1:** Proportion correctly answering knowledge questions (N = 310).

Questions	Correct response	Responding correctly (%)
It is not easy for a young women (under 20 years) to get pregnant	False	80
Douching/cleaning vagina after sex intercourse can prevent pregnancy	False	67
If the man pulls his penis out of my vagina before ejaculation, I will have no risk of getting pregnant	False	37
Using heroin or opioids can prevent pregnancy	False	37
If my partner is using heroin or opioids this can prevent pregnancy	False	36
Consistently and correctly using condoms is an effective method to prevent pregnancy	True	87
Consistently and correctly using condoms is an effective method to prevent HIV and other STIs	True	87

To assess consistent condom use, adolescents were asked whether they always, most of the time, sometimes, or never used condoms in the past month. This was followed with asking whether they had ever not used a condom while drunk in the past month. Consistent condom use was defined as always using condoms in the past month, including when drunk, and was determined separately for paying and non-paying partners. Dual method protection was defined as current use of any modern contraception plus consistent condom use with all sex partners in the past month. Unmet need for modern contraception was defined as not wanting to get pregnant and not currently using either modern contraception or condoms consistently in the past month. Adolescents were asked how many times they had been pregnant in their lifetime and how many of those pregnancies were unintended (mistimed or unwanted).

In this study, sexual partners were categorised as ‘non-paying’, including boyfriends, fiancés and husbands, or as ‘paying’, referring to regular or casual partners who had exchanged money or goods for sex. Alcohol use was dichotomised as daily or usual-drinker (more than twice a week) versus casual or non-drinker (once per week or less).

### Analysis

Analysis was conducted in Stata version 11. Univariable logistic regression was conducted to determine correlates of low knowledge, unmet need for modern contraception and experience of violence. All independent variables associated with outcome variables at *p<0*.*20* in univariable analysis were subsequently included in a multivariable logistic regression model; associations were considered significant in the multivariable model at *p<0*.*05*.

## Results

A total 378 adolescent FSWs were approached throughout all four urban areas of Kunming. Of these342 (90%) were eligible and consented to participate. Of these consenting participants, 310 (91%) completed the interview.

### Socio-demographic characteristics

Young female sex workers reported a mean age of 18.7years (standard deviation 1.2 years). Most (93%) relied on sex work as their main source of income, with a mean monthly income from sex work of RMB5000 (EUR 633), which is almost triple of an urban resident’s average monthly income Eighty-three percent (257/310) were internal migrants born outside Kunming. Most adolescents (86%) entered sex work in the past year. The median age of first sexual intercourse was 17 years (interquartile range [IQR]: 16–18) (Table 2).

**Table 2:** Socio-demographic characteristics and sex work characteristics of adolescent sex worker (N = 310).

Variables	% (n/N) or mean(SD) if stated
Age	
Mean years (SD)	18.7 (1.2)
Education level	
No school or primary school only	9% (27)
Middle school	74% (228)
High school	18% (55)
Current marital status	
Married or cohabitating	53% (165)
Current non-paying partner, not cohabitating	15% (46)
No current non-paying partner	32% (99)
Place of birth	
Kunming	18% (55)
Elsewhere	82% (255)
Currently living with	
Parents or relatives	6% (17)
Partner	24% (75)
Other sex workers or friends	39% (122)
Alone	31% (96)
Relationship duration with most recent (including current) non-paying partner	
≤1 years	65% (202)
>1 years	19% (59)
Never had a non-paying partner	15% (45)
Number of non-paying partners in past year	
None	18% (55)
One	51% (159)
Two or more	31% (96)
Experienced physical and/or sexual violence from any sexual partner in past year	
Yes	38% (118)
No	62% (192)
Number of paying partners in past month	
≤2	61% (189)
3 or more	39% (121)
Duration involved in sex work	
<1 month	12% (37)
1–6 months	50% (154)
7–12 months	25% (76)
>12 months	14% (43)
Any illicit drug use in the past year	
Yes	9% (27)
No	91% (283)

### Knowledge

SRH knowledge was poor in this group of adolescents (Table 1). The median number of correctly answered SRH questions was 4 (IQR: 3–6) out of 7. The median number of modern contraception methods participants had ever heard of was 3 (IQR: 2–4) out of 8 (Table 3). All adolescents had heard of condoms and 86% had heard of any other modern contraceptive method (most commonly emergency contraception [64%] or oral contraception [55%]). Adolescents reported accessing SRH information from peers (70%), traditional media (62%), the internet (37%), school classes (29%), health providers (27%), and parents (9%).

**Table 3:** Awareness and use of different types of contraception among adolescent female sex workers (N = 310).

Contraceptive methods	Heard of	Ever used^2^	Currently using^2^
	(%)n	(%)n	(%)n
Any modern contraception(including condoms)	100% (310)	99% (307)	93% (287)
Any modern contraception (excluding condoms)	86% (267)	57% (176)	28% (88)
Any LARC^1^	39% (120)	3% (9)	2% (5)
Female sterilization*	30% (92)	0.3% (1)	0.3% (1)
Male sterilization*	19% (58)	0% (0)	0% (0)
Oral Contraceptive Pill*	55% (172)	17% (53)	7% (21)
Injectable*	12% (38)	1% (4)	0.7% (2)
Implant*	6% (20)	0.3% (1)	0% (0)
IUD*	35% (107)	1% (4)	1% (3)
Diaphragm*	2% (6)	0% (0)	0% (0)
Emergency contraception	64% (198)	44% (135)	21% (64)
Condom	100% (310)	97% (302)	91% (281)
Condom use; Consistent	-	-	43% (132)
Dual method protection^+^	-	-	7% (23)
Any traditional method	67% (209)	54% (167)	40% (124)
Lactational amenorrhea	2% (6)	0.7% (2)	0.3% (1)
Rhythm	36% (111)	17% (52)	9% (28)
Withdrawal	44% (135)	29% (91)	20% (61)
Douching/cleaning after intercourse	35% (108)	26% (81)	6% (17)
Squat and push sperm out after intercourse	33% (101)	21% (64)	11% (34)

* included in ‘modern contraception’

^+^ Dual method protection defined as current use of any modern contraception plus consistent condom use with all partners in the past month

^1^ LARC (long acting reversible contraception) includes implant, injection, and IUD.

^2^ Women could report using multiple methods

Correlates of low SRH knowledge (scores below 8/15)in multivariable analysis were having experienced physical or sexual violence in the past year, inconsistent condom use with paying partners, and not obtaining SRH information from traditional media (Table 4).

**Table 4:** Factors associated with lower knowledge of sexual reproductive health among adolescent female sex workers (N = 310).

Factors	% (n/N) with low knowledge (score ≤ (n/N	Crude OR(95%CI)	p value	Adjusted OR (95%CI)	p value
All women	51 (158/310)				
Age, years					
15–17	45 (24/53)	1.0	0.36		
18–20	52 (134/257)	1.32 (0.73–2.38)			
Education level			0.31		
No school or primary school only	56 (15/27)	1.74 (0.69–4.40)			
Middle school	53 (120/228)	1.55 (0.85–2.80)			
High school	42 (23/55)	1.0			
Current non-paying partner			0.04		0.42
No	60 (59/99)	1.0		1.0	
Yes	47 (99/211)	0.60 (0.37–0.97)		0.77 (0.42–1.45)	
Experienced physical or sexual violence from any sexual partner (past year)			0.17		0.03
No	48 (92/192)	1.0		1.0	
Yes	56 (66/118)	1.38 (0.87–2.19)		1.84 (1.06–3.19)	
Average monthly income from sex work (Euro)			0.10		0.46
<633EUR	56 (79/141)	1.0		1.0	
≥633EUR	47 (79/169)	0.69 (0.44–1.08)		0.82 (0.48–1.39)	
Time in sex work			0.27		
<1 month	62 (23/37)	1.0			
1–6 months	47 (72/154)	0.53 (0.26–1.12)			
7–12 months	50 (38/76)	0.61 (0.27–1.36)			
>12 months	58 (25/43)	0.85 (0.34–2.08)			
Alcohol use, past year			0.36		
Abstainer or casual-drinker	45 (24/53)	1.0			
Daily or usual-drinker (more than twice a week)	52 (134/257)	1.32 (0.73–2.38)			
Abortion ever			0.08		0.55
No	47 (81/174)	1.0		1.0	
Yes	57 (77/136)	1.50 (0.95–2.35)		1.17 (0.69–1.99)	
Any self-reported symptom of STI (past year)			0.19		0.53
No	56 (59/105)	1.0		1.0	
Yes	48 (99/205)	0.73 (0.45–1.17)		0.83 (0.46–1.49)	
Received any SRH services^1^ in the past year			<0.05		0.20
No	77 (10/13)	1.0		2.51 (0.61–10.3)	
Yes	50 (148/297)	3.36 (0.91–12.4)		1.0	
Ever used any modern method of contraception (excluding condoms)^2^			<0.05		0.28
No	57 (77/134)	1.0		1.0	
Yes	46 (81/176)	0.63 (0.40–0.99)		0.73 (0.42–1.29)	
Currently using dual protection (modern contraception and condoms)^2^			0.04		0.24
No	53 (151/287)	1.0		1.0	
Yes	30 (7/23)	0.39 (0.16–0.99)		0.52 (0.17–1.56)	
Consistent use of condoms with non-paying partners (past month)^3^			<0.01		0.09
Inconsistent	54 (74/137)	1.0		1.0	
Consistent	34 (25/74)	0.43 (0.24–0.78)		0.56 (0.28–1.10)	
Consistent use of condoms with paying partners (past month) ^3^			<0.01		0.02
Inconsistent	65 (51/79)	1.0		1.0	
Consistent	46 (107/231)	0.47 (0.28–0.80)		0.50 (0.27–0.91)	
Obtained SRH information from health providers			0.84		
No	51 (116/226)	1.0			
Yes	50 (42/84)	0.95 (0.57–1.56)			
Obtained SRH information from traditional media(TV/movie/newspaper/magazine/book)			<0.001		<0.001
No	68 (80/118)	1.0		1.0	
Yes	41 (78/192)	0.33 (0.20–0.53)		0.35 (0.20–0.59)	
Obtained SRH information from Peers(classmate/colleague/friend/sexual partner)			0.03		0.05
No	60 (56/93)	1.0		1.0	
Yes	47 (102/217)	0.59 (0.36–0.96)		0.57 (0.32–1.00)	
Obtained SRH information from school			0.09		0.31
No	54 (119/220)	1.0		1.0	
Yes	43 (39/90)	0.65 (0.40–1.06)		0.74 (0.42–1.32)	
Obtained SRH information from the internet			0.46		
No	53 (102/194)	1.0			
Yes	48 (56/116)	0.84 (0.53–1.33)			
Obtained SRH information from parents/relatives			0.26		
No	52 (147/283)	1.0			
Yes	41 (11/27)	0.64 (0.29–1.42)			

^1^ SRH services including contraception, HIV/STI testing, free condoms

^2^ Dual protection defined as current use of any modern contraception plus consistent condom use with all partners in the past month. Modern contraception includes sterilisation, oral contraceptive pill, IUD, diaphragm, injection, emergency contraception, implant.

^3^ Consistent condom use was defined as always using condoms, including when drunk

### Contraception and condom use

Only 28% of respondents reported currently using a modern method of contraception, other than condoms, compared with 40% who were currently using a traditional method. Awareness and use of traditional methods was higher than awareness and use of LARCs (Table 3). Only 9 adolescents (3%) had ever used any form of LARCs. The most common current contraception used was condoms (91%), followed by emergency contraception (21%), and withdrawal (20%). Only 23 adolescents (7%) were currently using dual methods for contraception and prevention of sexually transmitted infections. Consistent condom use with all partners in the past month was reported by 43% (132/310).

Ninety-six (31%) participants reported more than one non-paying partner in the past year (Table 2). Of the 211 (68%) with a current non-paying partner, 74 (35%) reported consistent condom use with non-paying partners in the past month. However, when questioned further, 10 of these adolescents reported not using a condom in the past month while drunk. The most common justifications for inconsistent condom use with non-paying partners among the 147 adolescents who did so were use of other contraception (n = 39, 27%), partner refused to use condom (n = 39, 27%), forgot to use (n = 15, 10%), or did not use condom to show trust or not harm the relationship (n = 17,12%). Most (119/211, 56%) participants with a current non-paying partner reported that he was supportive of contraception, however, this did not seem to be associated with contraceptive use.

In the past month, median number of paying partners was 2 (IQR:1–6). Most (n = 231,75%) reported consistent condom use with paying partners in the past month. Commonly reported reasons for inconsistent condom use with paying partners among the 79 adolescents who did so were use of other contraception (n = 24, 30%), never using condoms with regular paying partners (n = 20,25%), client refused (n = 15,19%), or condoms were not available (n = 11, 14%).

At their first sexual intercourse, only 51 (16%) adolescents used either condoms or another modern contraceptive (47 used condoms and 4 used emergency contraception).

### Unmet need for modern contraception

Nearly all adolescents (n = 305, 98%) reported not currently wanting to get pregnant. Of these, 23 (8%) were currently using dual protection, 109(36%) reported consistent condom use with no other contraception, and 63(21%) used another form of modern contraception without consistent condom use. Therefore, unmet need for modern contraception was identified in 110 (35%) adolescents. In multivariable analysis unmet need was associated with having a current non-paying partner (adjusted odds ratio [AOR] = 3.41; 95% CI: 1.87–6.24), regular alcohol use (AOR = 3.19; 95%CI: 1.44–7.06), and having poorer SRH knowledge (AOR = 1.89; 95% CI: 1.13–3.15). (Table 5)

**Table 5:** Factors associated with current unmet need for modern contraception^1^ among adolescent female sex workers (N = 310).

Factors	Unmet need^1^	Crude OR	p value	Adjusted OR	p value
	% (n/N)	(95% CI)		(95% CI)	
TOTAL	35 (110)				
Age, years					
15–17	38 (20/53)	1.0	0.71		
18–20	35 (90/257)	0.89 (0.48–1.64)			
Education level					
No school or primary school only	33 (9/27)	0.95 (0.36–2.51)	0.95		
Middle school	36 (82/228)	1.06 (0.57–1.97)			
High school	35 (19/55)	1.0			
Current non-paying partner					
No	19 (19/99)	1.0	<0.001	1.0	<0.001
Yes	43 (91/211)	3.19 (1.81–5.64)		3.41 (1.87–6.24)	
Experienced physical or sexual violence from any sexual partner (past year)					
No	33 (64/192)	1.0	0.31		
Yes	39 (46/118)	1.28 (0.79–2.06)			
Average monthly income from sex work					
<633 Euro	37 (52/141)	1.0	0.63		
> = 633 Euro	34 (58/169)	0.89 (0.56–1.42)			
Average number of paying partners per week (past month)					
1–2	34 (65/189)	1.0	0.62		
3 or more	37 (45/121)	1.13 (0.70–1.82)			
Time in sex work					
<1 month	35 (13/37)	1.12 (0.44–2.84)	0.97		
1–6 months	36 (56/154)	1.18 (0.58–2.43)			
7–12 months	36 (27/76)	1.41 (0.52–2.52)			
>12 months	33 (14/43)	1.0			
Alcohol use, past year					
Abstainer or casual-drinker	17 (9/53)	1.0	<0.001	1.0	0.004
Daily or usual-drinker (more than twice a week)	39 (101/257)	3.17 (1.48–6.76)		3.19 (1.44–7.06)	
Abortion ever			0.86		
No	35 (61/174)	1.0			
Yes	36 (49/136)	1.04 (0.65–1.67)			
Any self-reported STI symptom of (past year)			0.02		0.30
No	27 (28/105)	1.0		1.0	
Yes	40 (82/205)	1.83 (1.10–3.07)		1.34 (0.76–2.35)	
Received any SRH services^2^ in the past year			0.10		0.08
No	15 (2/13)	0.32 (0.07–1.46)		0.24 (0.49–1.20)	
Yes	36 (108/297)	1.0		1.0	
Non-paying partners’ attitude towards contraception			0.93		
Non supportive	42 (35/83)	1.0			
Supportive	43 (53/124)	0.98 (0.56–1.71)			
SRH knowledge^3^			0.09		0.02
Low score	40 (67/169)	1.50 (0.93–2.40)		1.89 (1.13–3.15)	
High score	30 (43/141)	1.0		1.0	

^1^ Unmet need for modern contraception was defined as not currently intending to get pregnant and not using any modern contraception (sterilisation, oral contraceptive pill, intra-uterine device (IUD), diaphragm, injection, emergency contraception, or implant)

^2^ SRH services including family planning, HIV/STI testing, free condoms

^3^ Knowledge of sexual reproductive health was derived from a set of seven true/false questions plus one point for having heard of each of eight modern methods of contraception. Scores were split at the median (< = 7/15 vs>7/15)

### Pregnancy, abortion, and service utilisation

In total, 203 pregnancies among 144 adolescents were reported; the large majority (95%; 192) of these were unintended and 189 (93%) resulted in induced abortion. Of the remaining pregnancies, four were miscarriages, nine live births, and one stillbirth. Forty-one (13%) adolescents had more than one induced abortion in their lifetime (including 10 adolescents with 3 abortions and 1 adolescent with 4 abortions). Half (50%) of induced abortions were performed at a public hospital; the remainder were private hospitals/clinics (30%), family planning clinics (17%), or using take-home medication (3%). Of the 136 (44%) adolescents who had ever had an abortion, 74 (54%) reported ever experiencing complications, including menstrual disturbances (39; 29%), discharge (32; 24%), pain (29; 21%), fever and vaginal bleeding (5; 4%) and uterine perforations (1; 0.7%).

Two thirds (204; 66%) said they had received free condoms in the past year. Most (244/310, 79%) reported having received a medical consultation for HIV/STI in the past year, but only 140 (45%) reported an HIV test and 100 (71%) of these received their HIV test result. When asked what services and information they most wanted (multiple responses allowed), the most common responses were condoms (n = 189, 61%), HIV/STI information (n = 214, 69%), contraception information (42%), STI treatment (n = 129, 42%), reproductive health care (n = 139, 45%), free contraceptives (n = 65, 21%), and drug information (n = 40, 16%).

### Physical and sexual violence

Thirty-eight percent of adolescents (118/310) reported experiencing physical or sexual violence in the past year (Table 2). This included 98 adolescents experiencing physical violence and 75 experiencing sexual violence. Of these, eighty-eight (75%) had experienced violence perpetrated by non-paying partners and 72 (61%) by paying partners.

In the past year, those who had experienced violence were more likely to have had any STI symptoms (Odds ratio (OR) = 4.2; 95% CI: 2.4–7.5) and less likely to have received a consultation related to either HIV/STI (OR = 0.44; 95%CI 0.25–0.77) or contraception (OR = 0.47; 95% CI: 0.28–0.80).

## Discussion

This study is the first in China to describe SRH knowledge, unintended pregnancy and unmet need for modern contraception in a very marginalised and vulnerable group of adolescent female sex workers. We found that levels of SRH knowledge were low, use of contraception other than condoms was rare, that nearly half of the FSW had had an unintended pregnancy, and that 38% had experienced violence. Correlates of knowledge included having experienced physical or sexual violence in the past year, inconsistent condom use with paying partners, and not obtaining SRH information from traditional media; correlates of unmet need for modern contraception included having a current non-paying partner, regular alcohol use, and having poorer SRH knowledge; and correlates of violence included a history of STI symptoms and less service utilisation

Despite the majority of adolescent FSWs wanting to avoid pregnancy, the study demonstrated a high unmet need for effective contraception and a high prevalence of unintended pregnancy. Almost half of these adolescents reported a history of abortion, with 30% of those reporting multiple abortions which are slightly decreased in comparison with our 2010 study in same population (51% experiencing lifetime abortion and 41% of them reporting repeat abortions)[31], but still much higher than the prevalence of lifetime abortions among Chinese sexually-active girls aged 15–19 years in the general population (16% experiencing lifetime abortion and 5% of them reporting repeat abortions) [11].

With the exception of condoms and emergency contraception, current use of modern contraception was very low (<10%). None used reliable contraception at first intercourse. Similarly, our 2010 study showed reliance on inconsistent condom use and puts this population at risk of unwanted pregnancy, particularly with their non-paying partners (22% in both studies). While condoms are necessary to prevent HIV/STI, they are less effective at preventing pregnancy—particularly when used inconsistently as is the case in this sample. Almost half of all adolescent FSWs reported having ever used emergency contraception, and one in five reported current use. Access to emergency contraception in this population at high risk of unintended pregnancy is essential, particularly in the context of inconsistent condom use and sexual violence. However, emergency contraception has a high failure rate compared with other modern methods and is not a form of regular contraception, so the reliance on emergency contraception instead of use of more reliable methods is of concern[32]. Furthermore, 40% reported current use of less effective traditional methods to prevent pregnancy. Dual protection is the most effective way to prevent both unwanted pregnancy and HIV/STI, but reported current use of dual protection was very low in this group of young women (7%).

LARCs are highly effective and reliable contraceptive methods and are not dependent on individual compliance[33–35]. However, we found that just 39% had ever heard of any LARC method and only 2% were currently using one (1% for injectable, 0% for implant and 1% for IUD respectively). Our 2010 cross-sectional survey with a similar population showed similarly low uptake of LARC methods (2% for IUD and 0% for implants) [31]. This is also consistent with research among unmarried Chinese women which demonstrated low uptake of IUD and implants (0%–3%) [19]. Previous research has shown that increasing awareness of LARCs among both providers and young women may lead to initiating and using LARCs correctly and consistently over time [34,36]. This would include improving providers’ counselling skills to address fears and misinformation and promotion of LARCs and dual protection at all service delivery points including HIV and STI services, maternal health, and gynaecological clinics, as well as integrated into peer education and outreach programs targeting this population.

We found that SRH knowledge was poor in adolescent FSWs and misconceptions were prevalent; in addition a lower level of SRH knowledge was associated with not using dual protection, not using condoms with paying partners, and experiencing sexual partner violence. Our finding is consistent with a 2010 national representative survey, which showed only 3.2% migrant girls aged 15–19 years could answer five of five SRH knowledge questions correctly[11,12]. Previous research has revealed a very low level of SRH knowledge among unmarried migrant women (mean age = 20.2 years) due to poor SRH education provided for Chinese unmarried youth [16]. Despite our participants reporting having received SRH information from a range of sources, including school, traditional media, peers, family, and public or private SRH services. The majority of adolescent FSWs reported receiving some SRH services in the past year and almost half had attended a medical clinic for abortion. Interestingly, just 27% reported ever having received any information about contraception and SRH from health providers. Furthermore, those reporting receipt of information from health providers had the poorest level of knowledge, relative to those receiving information from other sources. These findings highlight a critical missed opportunity to provide essential SRH education in school and improve SRH services provision to this hard to reach population, and also suggests a need to improve the knowledge, attitudes and counselling skills of health providers.

Our study identified alcohol use as a risk factor for unmet need for modern contraception. Alcohol use is common in many sex work venues and in this sample was more common than other substance use. Sex workers are often pressured to promote the alcohol assumption for clients, or for them too as a potential source of income[37]. Previous studies show associations between alcohol use and HIV/STI, sexual risk behaviour and sexual violence among FSWs [27,28,37,38], and intoxication in both FSWs and their clients can increase the difficulty of negotiating condom use. Clustering of risk factors is common in adolescence[39], and our findings have emphasised the need to address the complex determinants of risky sexual behaviour, rather than relying on single-focused interventions. In South Africa and Kenya, interventions to provide skills training to mitigate alcohol-related risk and better coping with intoxication at work among FSWs were associated with increased condom use[37,40].

There is strong evidence that SRH and inconsistent condom use are affected by gender-based violence[25,41,42]. We found that 38% adolescent FSWs had experienced recent physical or sexual violence from sexual partners. This was associated with lower SRH knowledge and poorer service utilisation. Research with other Chinese FSWs has found the proportion ever experiencing violence to range from 16% to 58%[43–45]. Sexual and gender-based violence against FSWs is a pervasive and complex issue, and addressing this requires input from multiple sectors including the community, health, police and legal sectors. Interventions to prevent and protect from violence may include empowering FSW with knowledge about their rights and skills for negotiation, self-protection and strategizing responses training, peer support and information sharing, promoting workplace security, provision of legal support, public advocacy, and supportive legislation[37,46–48]. In addition, health care providers and others working with FSW should be trained to refer victims of violence to appropriate health, psychosocial, and legal support. There is also a need for training in skills for condom/contraception negotiation among these adolescent FSWs, as partner refusal to use condoms was common.

Stigma and the illegal status of sex work, judgemental provider attitudes, and inadequate counselling skills all hinder comprehensive SRH information provision from public health sectors [11,12,14,49,50]. Furthermore, discriminatory policy and regulations can present significant barriers to this population, many of whom face the triple stigma of being unmarried, engaged in sex work, and of migrant status. Government-funded family planning clinics in China provide a full-range of contraceptives at a very low cost. However, current policies require parental consent and ascertainment of marital status and residency, and prevent unmarried women or adolescent girls’ access to these services. Additionally, most adolescents surveyed were internal migrants; current policy requires identification for medical insurance and social security, and places some limitations on these services for migrants. Given their criminalised and highly mobile status, adolescent FSWs may be reluctant or have more difficulties to obtain the medical insurance, and therefore have limited access to public health care. Targeted outreach services may help overcome some of these considerable challenges and can also provide a link between marginalised young people and mainstream services. Peer support, mobile clinics and other outreach programs in Lao PDR, Thailand and the Philippines have successfully reached young marginalised people, include sex workers, with information, counselling and services and demonstrated improved knowledge, self-esteem and use of contraception and condoms[51–53]. The Ministry of Health’s 2012 China Country Progress Report and our previous research[27] demonstrated high coverage (more than three quarters) of HIV/STI outreach programs in this population, therefore integrating contraception information and services into this existing platform may help address adolescent FSWs’ unmet needs.

In this study, adequate pre-communication between the study team and the gatekeepers of entertainment establishments facilitated the recruitment procedure. In comparison to our 2010 study[27] with a similar population in Kunming, the number of eligible subjects identified increased (295 vs 342) and the refusal rate was reduced (22% vs 10%). The random sampling method increased representativeness to the study population. However, our study has several limitations. The major limitation of the study was its cross-sectional design, meaning that causality cannot be attributed. Secondly, data were self-reported and may have been subject to recall or social desirability bias. Thirdly, reasons for non-use and discontinuation of contraceptives, preferred methods of contraception, and reproductive outcomes (including pregnancy) before and after entering sex work require further investigation. Further, our study is limited to Kunming city, similar research is urgently needed in other regions of China among similar populations. Lastly, the different measures of SRH knowledge used in different studies with adolescents in diverse populations raised the difficulty to compare levels of knowledge. The findings of this study could inform selection and standardization of key indicators of knowledge of sexual and reproductive for female adolescents in the future.

In conclusion, the high level of unmet need for modern contraception, high prevalence of unintended pregnancy and abortion, and low levels of SRH knowledge among FSWs in this study demonstrate the urgent need for comprehensive and accessible SRH services for adolescent sex workers in Kunming, China.
